# Development and feasibility testing of decision support for patients who are candidates for a prophylactic implantable defibrillator: a study protocol for a pilot randomized controlled trial

**DOI:** 10.1186/1745-6215-14-346

**Published:** 2013-10-22

**Authors:** Sandra L Carroll, Michael McGillion, Dawn Stacey, Jeff S Healey, Gina Browne, Heather M Arthur, Lehana Thabane

**Affiliations:** 1 School of Nursing, Faculty of Health Sciences, McMaster University, Hamilton, ON, Canada; 2 Population Health Research Institute, Hamilton Health Sciences, Hamilton, ON, Canada; 3 Heart and Stroke Foundation/Michael G DeGroote Endowed Chair in Cardiovascular Nursing, Hamilton, ON, Canada; 4 School of Nursing, University of Ottawa, Ottawa, ON, Canada; 5 University Research Chair in Knowledge Translation to Patients, Ottawa, ON, Canada; 6 Ottawa Hospital Research Institute, Ottawa, ON, Canada; 7 Hamilton Health Sciences, Hamilton, ON, Canada; 8 Department of Medicine, McMaster University, Hamilton, ON, Canada; 9 Department of Clinical Epidemiology and Biostatistics, McMaster University, Hamilton, ON, Canada; 10 Departments of Clinical Epidemiology and Biostatistics, Pediatrics and Anesthesia, McMaster University, Hamilton, ON, Canada; 11 Biostatistics Unit, St Joseph’s Healthcare - Hamilton, Hamilton, ON, Canada

**Keywords:** Decision aid, Feasibility trial, Implantable defibrillator, Decision-making, Shared decision-making

## Abstract

**Background:**

Patients, identified to be at risk for but who have never experienced a potentially lethal cardiac arrhythmia, have the option of receiving an implantable cardioverter defibrillator (ICD) as prophylaxis against sudden cardiac death - a primary prevention indication. In Canada, there is no clear framework to support patients’ decision-making for these devices. Decision support, using a decision aid, could moderate treatment-related uncertainty and prepare patients to make well-informed decisions. Patient decision aids provide information on treatment options, risks, and benefits, to help patients clarify their values for outcomes of treatment options. The objectives of this research are: 1) develop a decision aid, 2) evaluate the decision aid, and 3) determine the feasibility of conducting a trial.

**Methods/design:**

A development panel comprised of the core investigative team, health service researchers, decision science experts, cardiovascular healthcare practitioners, and ICD patient representatives will collaborate to provide input on the content and format of the aid. To generate probabilities to include in the aid, we will synthesize primary prevention ICD evidence. To obtain anonymous input about the facts and content, we will employ a modified Delphi process. To evaluate the draft decision aid will invite ICD patients and their families (n = 30) to rate its acceptability. After we evaluate the aid, to determine the feasibility, we will conduct a feasibility pilot randomized controlled trial (RCT) in new ICD candidates (n = 80). Participants will be randomized to receive a decision aid *prior* to specialist consultation *versus* usual care. Results from the pilot RCT will determine the feasibility of research processes; inform sample size calculation, measure decision quality (knowledge, values, decision conflict) and the influence of health related quality of life on decision-making.

**Discussion:**

Our study seeks to develop a decision aid, for patients offered their first ICD for prophylaxis against sudden cardiac death. This paper outlines the background and methods of a pilot randomized trial which will inform a larger multicenter trial. Ultimately, decision support prior to specialist consultation could enhance the decision-making process between patients, physicians, and families, associated with life-prolonging medical devices like the ICD.

**Trial registration:**

ClinicalTrials.gov: NCT01876173

## Background

Sudden cardiac death due to ventricular tachyarrhythmia is a serious cause of cardiovascular death in Canada [1], Europe [2,3], and the United States [4]. An innovative cardiovascular intervention to reduce the incidence of sudden cardiac death is the implantable cardioverter defibrillator (ICD). To restore a normal heart rhythm, the ICD delivers an internal shock, or anti-tachycardia pacing via an internally placed cardiovascular lead attached to the right ventricle of the heart. Evidence from randomized controlled trials (RCTs) and systematic reviews has demonstrated the effectiveness of ICDs for improving mortality and morbidity outcomes in patients with ischemic and non-ischemic disease, and heart failure [5-14]. Clinical practice guidelines provide current risk criteria to guide patient ICD candidacy [15]. Patients with or without ischemic disease, and reduced left ventricular function (among other criteria) may be offered an ICD for a primary prevention indication or sudden death prophylaxis. In addition, patients with heart failure may also be eligible for an ICD with the option of cardiac resynchronization, to augment ventricular efficiency and output [16]. Furthermore, indications for ICDs are broadening to include prophylaxis for genetically inherited channelopathies such as long QT-syndrome [16]. Estimates of the number of eligible Canadians range from 85,000 to 92,000 [16,17], with 3,700 candidates accruing annually.

Specialized electrophysiologists navigate the evidence associated with device options and risk-stratify their patients. However, for the patient and their families, the decision to receive an ICD may not be straightforward. Additional complexities surrounding ICD implantation, benefits, complications, and delivery of therapy exist, which warrant special attention to prepare patients to make informed, value-based choices. ICDs have benefits and risks associated with implantation, replacement, advisories, and psychosocial adjustment during the ICD lifetime [18-22]. These complex factors, balanced with the presence of competing mortality risks warrant structured, evidence-informed decision support. Decision support using a patient decision aid (PtDA) can moderate decisional conflict or treatment uncertainty and is used successfully in practice among several health treatment situations to improve patient outcomes [23]. A PtDA uses informational documents, or visual aids designed to *prepare* patients for decision-making about treatment options. Decision aids are intended to supplement rather than replace counselling from practitioners [24]. They are not passive informed consent materials, or educational interventions that are not geared to a specific decision. A 2011 Cochrane review of 86 studies found that when patients use decision aids they: a) improve their knowledge of the options; b) are helped to have more accurate expectations of possible benefits and harms; c) reach choices that are more consistent with their informed values; and d) participate more in decision making [23]. To date, the effectiveness of decision aids use in cardiovascular patients has been tested in atrial fibrillation [25,26], ischemic heart disease [27], and angiography [28,29]. To our knowledge, there is no research evaluating the use of a PtDA in patients who are offered an ICD for prophylaxis against sudden cardiac death in Canada.

Decision aids are used when there is more than one option. In the context of ICDs, this entails a choice between an elective prophylactic intervention versus continued optimal medical management - not *death,* but an *annual risk* of an arrhythmia. However, candidates who live with advancing heart failure may not comprehend the implications of living longer with their heart failure or the risks associated with ICD therapy if device shocks are received. At the point of decision-making for life-prolonging interventions, provision of time to consider end-of-life decisions may seem to be a contradiction. Nonetheless, this conversation and an introduction to what will be a future health decision are entrenched in the choice to receive an ICD [30]. Decision aids are embedded within the framework of shared decision-making, wherein together, patient, families, and physicians reach agreement on treatment-related decisions [31]. This approach stems from the concept of patient centered care [32]. In essence, shared decision-making encourages *active* engagement of patients during the process of decision-making and decision aids facilitate their participation. We have identified a need to provide better decision support for ICD patients during their decision-making process [30].

A considerable body of evidence gathered from ICD recipients suggests that psychosocial factors, including depression [33], anxiety [34-37] and personality [38,39] are associated with patient reported outcomes. Patients who receive shocks from the ICD report reductions in health-related quality of life (HRQL) [40,41]. Furthermore, the prevalence of depression or depressive symptoms is high both pre-ICD implantation and post-ICD implantation [42]. The influence of these factors at the time of health care decision-making is not known. We previously reported a high prevalence of depressive symptoms pre-implantation (30%), accompanied by poor pre-implantation mental-HRQL [43]. Poor mental-HRQL had a significant influence on early ICD acceptance in participants. This work highlights patient-specific factors known to influence patients’ HRQL, acceptance of, and adjustment to living with an ICD. Furthermore, a standard ICD does not alter or improve a patient’s underlying cardiovascular condition or symptoms. However, an ICD with added cardiac resynchronization (CRT) has a greater likelihood of improving heart failure symptoms and HRQL due to its ability to restore synchrony of the ventricles [11]. Differentiating what an ICD can and cannot do is vital for patients to consider at the time of decision-making.

### Conceptual framework

The Ottawa Decision Support Framework (ODSF) [44] provides the conceptual framework for this research. The ODSF is rooted in evidence, concepts, and theories from psychology, decision analysis, decisional conflict, values, and self-efficacy [45]. Importantly, the ODSF emphasizes decision *preparation* using a framework that separates the effects of each decision support method [44]. The framework has three main elements (decisional needs, decision support, and decision quality) [45]. The framework asserts that participant’ decisional needs (perceptions of the decision, knowledge, unrealistic expectations of outcomes, uncertainty, values, support, and resources, personal and clinical characteristics), affect decision quality. Decision quality is defined as reaching a decision that is based on the best available evidence and patients’ informed values for outcomes of options. The second element; decision quality, affects behavior (for example, delays), health outcomes, emotions, costs, and use of services [45]. Clinical characteristics may include the health status of patients. The final element is decision support [45]. The aim of decision support when delivered through a decision aid, counselling, or coaching is to improve decision quality by addressing unresolved decisional needs. Treatment effects associated with decision support, framed within the ODSF have been empirically tested [44,46-51].

## Methods

To meet the objectives of this research, the study will take place in three steps using a combination of quantitative and qualitative methods. In step one, we will draft, assess, and revise the decision aid contents using an inter-disciplinary panel of health services researchers, educators, practitioners, and ICD patients. In step two, we will invite patients and their family members who are experienced with the decision (have an ICD) to review and give feedback on the draft PtDA. Modifications based on patient and family feedback, will be made if necessary. Step three will employ a feasibility pilot randomized controlled trial in a single center, employing 1:1 allocation. The study setting is Hamilton Health Sciences (HHS), an academic tertiary care centre in Hamilton, ON, Canada. The center serves over one point four million residents in the regions of Brant, Burlington, Haldimand, Hamilton, Niagara, and Norfolk for cardiovascular procedures.

### Step one: development of the decision aid

Development of the PtDA is guided by the ODSF and International Patient Decision Aid Standards (IPDAS) quality criteria [24,45]. Development includes, 1) synthesize evidence- based information about ICDs, 2) consider ways of presenting the ICD options (accepting or declining an ICD) ensuring good risk communication and addressing uncertainties, 3) determine the values associated with the benefits and risks that are important to patients, and 4) draft decision aid using a structured approach that can guide the patient through the decision-making process. In addition to IPDAS, development of the PtDA will be guided by components suggested in O’Connor and Edwards [52], and O’Connor and Jacobson [51].

A development panel comprised of the core investigative team, cardiovascular healthcare practitioners (heart failure, general cardiology, internal medicine, and nurse specialists), health service researchers, ICD patient representatives, and a decision aid expert, will collaborate to provide input on the information, content, and format of the aid. Early engagement of patient representatives and knowledge users is intentional as an integrated knowledge translation technique in order to build a foundation for effective knowledge translation and user uptake of the decision aid in a clinical context [53].

In order to inform the risk and benefit information for the ICD decision aid, the we will adapt and synthesize existing ICD practice guidelines, current meta-analyses of randomized trials, and systematic reviews specific to the primary prevention ICD indication population to develop probabilities to include in the aid [14,20,22,54-56].

The process of selecting final content to include in the ICD PtDA will include a survey using a Delphi process [57,58]. Electrophysiologists, nurses, decision science experts, stakeholders, and patients with ICDs will be invited to provide feedback on PtDA content. We will employ an electronic survey, utilizing confidential login procedures through a secure website administered at the study site. If requested by patient participants, anonymous paper versions will be provided. The Delphi process will seek to overcome some of the challenges found with making decisions in groups. The Delphi approach attempts to assess the extent of agreement and to resolve disagreement. The features include: 1) anonymity; achieved by use of a questionnaire, 2) iteration where the processes occur in ‘rounds’, 3) controlled feedback*,* showing the distribution of the group’s response, and 4) statistical group response using summary measures [57,58]. The process will take place in up to three rounds. Round 1: participants will be invited to provide opinions on the a) facts and information about ICDs, b) presentations of risks and benefits, and c) values clarification exercises, based on knowledge and experience. The opinions will be grouped under discreet headings drafted for circulation to all participants on a questionnaire; round 2*:* participants rank their agreement with statements that did not reach agreement in round 1; the rankings will be summarized and included in round 2. If agreement is not reached in round 2, the process will be repeated for a final round.

### Step two: preliminary acceptability testing in patients with ICDs

To ensure the decision aid is acceptable, simple to complete, and clearly formatted for patient use, we will use a convenience sample of 30 patients from HHS with ICDs (primary prevention indication), and family members involved with the decision, to review the PtDA. In 2011, HHS received approximately 362 new ICD patient referrals. We will invite patients who are within one to six months of receiving the ICD. These patients will have the requisite experience with making ICD-related decisions, critical for informed and comprehensive assessment of the acceptability, feasibility, and formatting of the decision aid [59]*.* Exclusion criteria include 1) inability to understand the decision aid due to a language or visual impairment or 2) unstable cardiac condition. During scheduled outpatient ICD clinics, a physician or nurse specialist who is part of the healthcare team will introduce the study to patients. Patients who agree will receive an information package in the mail or if preferred, face to face. Demographics and medical history will be collected from the patients’ medical record and acceptability measures completed (see below).

### Step two: decision aid acceptability outcomes

Participants will complete the Decision Support Acceptability Scale (DSTA). The tool is used frequently during decision aid development and preliminary evaluation [60]. The DSTA questionnaire is comprised of ten items addressing comprehensibility of the decision aid components, balance in the presentation of options, the amount of information, and overall suitability [60]. Responses will be reported descriptively in terms of proportions of positive or negative responses to each item. We will summarize patient and family member feedback, any issues for discussion or revisions will be brought to the development panel.

### Step three: feasibility RCT testing

#### Primary purpose

##### Feasibility

**Figure 1:**
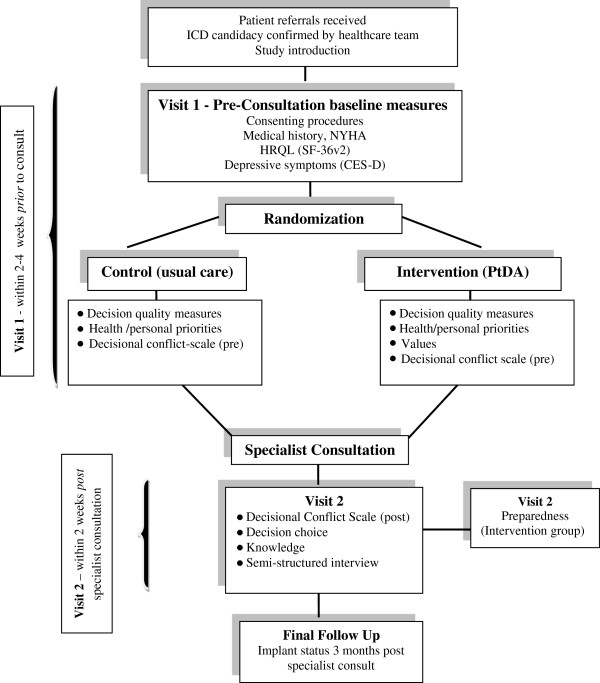
Feasibility pilot randomized controlled trial.

Guided by the Ottawa Decision Support Framework [45] and the UK Medical Research Council recommendations [61], we will employ a feasibility pilot RCT (see Figure 1).

Feasibility endpoints will focus on *processes* (referral, recruitment rates, successful delivery of the decision aid, randomization procedures, the monitoring of *resources* (budget), and study *management* (trial coordination, human resources) [62]. We will assess the proportion of patients who complete the decision aid, percent of missing data, and questionnaire completion rates. Criteria for assessing the success of feasibility on key process outcomes include: 1) participant recruitment rate of at least 80% of all eligible patients, 2) successful delivery and completion of PtDA in 80% of consented patients prior to specialist consultation, 3) percentage of patients who complete PtDA of at least 80%, and 4) questionnaire completion rate of at least 80%.

### Feasibility methodology

#### Participants, setting, and sample size

Participants will be patients referred for consultation for *de novo* ICDs for prophylaxis against sudden cardiac death. Exclusions are: 1) unable to understand the decision aid due to a language barrier or visual impairment, and 2) referral for an ICD with cardiac resynchronization or secondary prevention indication. To assess the feasibility of a conducting a larger trial, we will enroll 80 patients (40 per arm) over 10 months. The sample size for this pilot trial was based primarily on feasibility considerations. This pilot RCT will inform the estimates of effect sizes and variance for a larger trial.

### Baseline visit

Members of the healthcare team will introduce the study to patients after ICD candidacy is determined but prior to their consultation in which the decision may be discussed/made. Introduction of the study will take place by mail or phone after candidates are contacted by the health care team to confirm the consultation appointment. Following the introduction to the study, the research assistant (RA) will either mail patients a study package, or arrange to meet patients for a baseline visit prior to the specialist consultation visit. At the time of the baseline visit, the RA will review study procedures, and obtain written consent. Following consent, the RA will collect baseline demographics, medical history, heart failure classification (NYHA), HRQL, and depressive symptoms. Once complete, the RA will randomize the patient.

### Allocation

We will randomly allocate patients to intervention (PtDA *prior* to specialist consult) or usual care. We will use a secure electronic randomization system (http://www.randomize.net/). The allocation sequence is concealed. The research assistant will enroll participants and access the randomization system to determine group assignment following baseline data collection (see below).

### Blinding

Because of the nature of the intervention, patients assigned to PtDA, and the RA, will not be blind (to decision aid or usual care). Data analysts will be blind to treatment assignment for outcomes measured in both groups (knowledge, HRQL, preference, health/personal priorities, decisional conflict pre/post, and decisional outcomes). Participants and the RA will not be aware of treatment assignment during baseline data collection.

### Intervention

The intervention group will receive and use the PtDA with a trained research assistant *prior* to specialist consultation.

### Usual care

The usual care group will receive standard HHS information upon arrival for their specialist consultation.

### Visit one - pre consultation

#### Baseline measurements - (intervention and usual care)

To measure baseline psychosocial status and HRQL, we will use The Medical Outcomes Trust Short Form (SF-36v2), a generic HRQL scale used extensively in health care and ICD research. The reliability and validity of the SF-36v2 is well established [63]. The Center for Epidemiologic Studies Depression Scale will measure depressive symptoms (CES-D) [64]. The CES-D has demonstrated good reliability and validity across different community and clinical settings [65]. The NYHA [66] classifies functional abilities based on self reported symptom limitations; it has strong predictive utility and is used widely in practice. Participants will also rank their five priorities (health and personal).

Patients randomized to the intervention group will complete the PtDA and decision quality measures (values, decisional conflict, and knowledge). The usual care group will complete decision quality measures (decisional conflict, knowledge). Both groups will complete these measures prior to specialist consultation if feasible.

### Visit two - post consultation

After confirming completion of the specialist consultation, the RA will contact participants for visit two. We will measure decisional conflict and knowledge in both groups (within two weeks post-consultation). Preparation for decision-making will be measured in the intervention group. Where feasible, and participants are in agreement, the RA will arrange to conduct a brief semi-structured interview to gain additional qualitative feedback about the PtDA and process*.*

### Feasibility outcomes and measures

The primary feasibility outcomes will focus on *processes* (referral rates, recruitment rates, successful and timely delivery of the decision aid, randomization procedures, the monitoring of *resources* (budget), and study *management* (trial coordination, human resources) [62] (see above).

### Decision quality measures (knowledge, values, preferences, decisional conflict)

To measure patients’ values, we will ask patients (intervention group) to rate the personal importance of benefits, harms, and preferences using a rating scale (0 to 10). Values item selections will be developed by the core panel based on ICD evidence [30,67], the Delphi, and clinical expertise. We will also measure all patients pre-consultation preferences (get a device, don’t get a device). Knowledge will be measured both pre and post consultation in both groups using five questions related to treatment, benefits, and risks. Initial reliability and validity testing of the knowledge test will take place during the feasibility testing. Finally, to measure decisional conflict, we will use the Decisional Conflict Scale [68,69]. The scale measures a person’s perception of the difficulty in making a decision, the extent to which they feel uncertain about treatment options, are knowledgeable about the risks and benefits of options, clear about personal values, and supported in decision making [68]; the scale has demonstrated good reliability (Cronbach’s alpha coefficients > 0.78) and predictive validity [69]. Higher scores indicate greater decisional conflict and patients experiencing decisional conflict are more likely to change their minds, delay decisions, express regret, and fail knowledge tests [69,70].

### Decision preparation scale

Preparedness (intervention group), will be measured using the Preparation for Decision Making scale [59,71,72] which has ten items assessing patient’s perceptions of how useful a decision aid is in preparing them to communicate with their practitioner during consultation. The scale has demonstrated good internal consistency reliability (Cronbach’s alpha 0.92 to 0.96).

### Evidence to inform the calculation of a sample size for a larger trial

The pilot RCT will inform estimates of effect sizes and variance for a larger trial. More specifically, we will identify clinically significant differences in the change scores of decision quality measures (Decisional Conflict) that correspond to acceptable levels of patient uncertainty about their course of action (treatment options).

### Final follow-up

Three months after participants complete their baseline visit 1, the RA will confirm vital status, and device implant status from the health record.

### Data collection, management and analysis

Results for feasibility outcomes including participant recruitment, referral rates, patient uptake of the decision aid, and missing data will determine feasibility of study processes. This will mainly be descriptive in nature. Continuous variables will be summarized using descriptive statistics and measures of central tendency (means, standard deviations) and categorical variables using percentages and frequencies. Assumptions of the normality of data will be assessed. When normality assumptions are not met, non-parametric tests will be applied [73]. To determine differences in decision quality (Decisional Conflict) each group’s scores will be compared using independent sample *t*-test at two time points. The first comparison will be within group (pre and post consultation) followed by 2) between group differences in the change scores using analysis of covariance (ANCOVA) that adjust for baseline scores [73]. All quantitative tests will be two-sided and conducted at the 5% level of significance. We will also use knowledge scores > 66% to predict decision choice using logistic regression techniques. To examine associations between baseline HRQL, depressive symptoms, and 1) decision choice, and 2) values, we will use regression analysis and calculation of odds ratios and 95% confidence intervals where appropriate. SAS 9.2 (Cary, NC, USA) will be used to conduct quantitative analyses.

Qualitative data (interviews, field notes) will be analyzed using interpretive descriptive analysis [74-76]. Interviews will be recorded, transcribed verbatim, and anonymized. NVivo (http://www.qsrinternational.com/contact.aspx) software will assist with the management and coding of findings. We will analyze patient interviews to identify themes surrounding patient perceived barriers to decision aid uptake.

### Data management

To ensure data quality, all data will be collected via standardized forms and entered electronically via facsimile transmission to the data coordinating center using TeleForm (http://www.cardiff-teleform.com). All original paper forms will be stored at the study site (McMaster). Data integrity will be enforced by a variety of mechanisms. Data discrepancies and inquiries will be addressed individually and responses to each inquiry generated by the study site. Study data will be stored in locked cabinets. Access to data will be restricted.

### External review of the PtDA

The decision aid will be uploaded for review via the Ottawa Decision Aid Library Inventory and, clinical experts external to the development process will be invited to review the decision aid as per IPDAS.

## Discussion

This paper describes the protocol for the development of and feasibility testing of a decision aid for patients who are candidates for their first ICD as prophylaxis against sudden cardiac death associated with cardiac arrhythmias. Previously, in the Canadian ICD population, a need for decision support was revealed [30], thus the goal of this work is to develop this support. ICDs are life-prolonging medical devices and are an important cardiovascular treatment option for patients who are at risk for sudden cardiac death. Therefore, the provision and availability of decision support is imperative for patients and families to facilitate quality decision making (informed, deliberate, and value-based). We are developing a PtDA for a standard ICD when it is offered for a primary prevention indication. In Canada, this is an elective procedure whereby patients are referred to centers who offer specialized electrophysiology services. It is our intention to deliver the PtDA intervention *prior* to specialist consultation, thus the feasibility component of this work is significant for a future multi-center trial. To our knowledge, the provision of a PtDA prior to specialist consultation has not been tested in Canada.

A standard ICD cannot offer patients the potential to improve heart failure symptoms found with devices that have added cardiac resynchronization (CRT). It is important that patients who prioritize symptom reduction as an important health goal, who are not eligible for added CRT, understand this difference at the point of decision-making. An important ICD fact we will introduce in our PtDA is the potential for future deactivation of the ICD (or alternatively the option *not* to replace the ICD when the device battery approaches its end). Understanding long-term factors associated with an intervention such as the ICD early in the trajectory may assist patients and families with advanced directives planning where appropriate [77]. Moreover, we are engaging experienced ICD patients in the process of our PtDA development to ensure that our work is patient oriented and reflects the views of patients who are familiar with making this decision. Finally, we will determine the feasibility of conducting a larger trial. It is our goal to disseminate the PtDA and make it assessable to the public.

### Ethics, consent, and dissemination

The study protocol (version 1; 20 March 2012) was approved by the Hamilton Integrated Research Ethics Board (#12-214), and registered at ClinicalTrials.gov. (NCT01876173). Informed consent will be obtained from all participants by a research assistant who is not involved with direct care in step two and step three of the study. Each participant will receive a signed copy of the consent/information sheet. If important protocol modifications are made communication to relevant parties (for example, investigators, Ethics Board, trial registry) will be undertaken. All personal information about potential and enrolled participants when stored electronically will be encrypted and password protected. Study data collection forms will not contain any personal identifiers, only study identification numbers. Participants in steps two and three will receive a gift card as a token of appreciation.

## Trial status

Currently enrolling patients.

## Abbreviations

ANCOVA: Analysis of covariance; CES-D: The center for epidemiologic studies depression scale; CRT: Cardiac resynchronization therapy; DSTA: Decision support acceptability scale; HRQL: Health-related quality of life; HHS: Hamilton health sciences; ICD: Implantable cardioverter defibrillator; IPDAS: International patient decision aid standards; NYHA: New York heart association; ODSF: Ottawa decision support framework; PtDA: Patient decision aid; RA: Research assistant; RCT: Randomized controlled trial; SF-36v2: The medical outcomes trust short form scale.

## Competing interests

The authors of this protocol disclose no financial conflict or competing interests for this study.

## Authors’ contributions

SLC participated in the design of the study, wrote the first draft of the manuscript, and applied for funding. SLC, MM, DS, JH, LT, HMA, GB participated in the design of the study, PtDA development panel, and critical review of this manuscript. All authors read and approved the final manuscript.
